# Expression of genes encoding extracellular matrix proteins: A macroarray study

**DOI:** 10.3892/or.2014.3493

**Published:** 2014-09-17

**Authors:** KONRAD FUTYMA, PAWEŁ MIOTŁA, KRYSTYNA RÓŻYŃSKA, MAŁGORZATA ZDUNEK, ANDRZEJ SEMCZUK, TOMASZ RECHBERGER, JACEK WOJCIEROWSKI

**Affiliations:** 1 Second Department of Gynecology, Medical University of Lublin, Lublin, Poland; 2 Department of Clinical Genetics, Medical University of Lublin, Lublin, Poland; 3 Department of Clinical Pathology, Medical University of Lublin, Lublin, Poland; 4 Department of Medical Genetics, Medical University of Lublin, Lublin, Poland

**Keywords:** endometrial cancer, cDNA macroarrays, fibronectin, osteonectin, extracellular matrix proteins

## Abstract

Endometrial cancer (EC) is one of the most common gynecological malignancies in Poland, with well-established risk factors. Genetic instability and molecular alterations responsible for endometrial carcinogenesis have been systematically investigated. The aim of the present study was to investigate, by means of cDNA macroarrays, the expression profiles of genes encoding extracellular matrix (ECM) proteins in ECs. Tissue specimens were collected during surgical procedures from 40 patients with EC, and control tissue was collected from 9 patients with uterine leiomyomas. RNA was isolated and RT-PCR with radioisotope-labeled cDNA was performed. The levels of ECM protein gene expression in normal endometrial tissues were compared to the expression of these genes in EC specimens. Statistically significant differences in gene expression, stratified by clinical stage of the ECs, were detected for aggrecan, vitronectin, tenascin R, nidogen and two collagen proteins: type VIII chain α1 and type XI chain α2. All of these proteins were overexpressed in stage III endometrial carcinomas compared to levels in stage I and II uterine neoplasms. In conclusion, increased expression of genes encoding ECM proteins may play an important role in facilitating accelerated disease progression of human ECs.

## Introduction

Endometrial cancer (EC) is the most common gynecological malignancy in Poland, with nearly 5,500 new cases a year and well-established risk factors (1,2). The majority of cases are designated as type I estrogen-dependent tumors according to the Bokhman’s dualistic model of endometrial tumorigenesis (3). Another 10–20% of uterine malignancies, designated as type II carcinomas, follow the estrogen-unrelated pathway and arise in the background of atrophic endometrium (4,5). Genetic instability and molecular alterations have been systematically investigated in ECs, and it is known that four major genetic changes are generally responsible for endometrial carcinogenesis: silencing of the *PTEN* tumor-suppressor gene, presence of microsatellite instability due to alterations of the mismatch repair genes, mutation of the *K-ras* proto-oncogene and alteration of the *β-catenin* gene. Subsequent steps in studying the molecular aspects of carcinogenesis should be carried out with the aim to provide a better understanding of the influence of known gene mutations on expression of other genes as well as on metabolic processes that have not yet been investigated (6–9).

The invasive phenotype is crucial for the ability of cancer cells to infiltrate the surrounding tissue and to metastasize. During neoplastic transformation, cancer cells cross the basement membrane (BM), the extracellular matrix (ECM) and vessel walls (10,11). ECM components such as glycoproteins, proteoglycans and other proteins responsible for cellular signaling play a crucial role in carcinogenesis (12,13). The most important changes during carcinogenesis are observed in the BM. The BM separates from the stroma which leads to defects in its continuity (14). BM is a layer 50–100 nm thick, composed of different proteins (collagen type IV, laminin, nidogen and perlecan). Collagen type IV and laminin are responsible for stabilization of the BM, whereas nidogen and perlecan play a crucial role in extracellular signaling. In addition to those four major proteins, many other proteins are involved in forming the BM, for example: fibronectin, fibulin, agryne, tenascins, other types of collagen and osteonectin, all of which are responsible for ECM specificity (15–21).

Advancement in molecular genetics in the field of microarrays and macroarrays has made the analysis of gene interactions possible. Techniques based on the large scale measurement of mRNA expression can be easily applied for evaluating the complexity and influence of gene expression on cellular metabolism and processes leading to neoplastic transformation (22,23). Continual advancement of such techniques, along with rigorous analysis, is the key to identifying the most relevant genetic changes and correlating them with patient outcome. Such research is crucial for the development of tests with the ability to detect cancer early, as well as for the treatment of EC (24–26).

The aim of the present study was to investigate the expression profiles, as measured by cDNA macroarrays, of genes coding for ECM proteins in EC.

## Materials and methods

### Study group

The present study was approved by the local ethics committee, and all patients signed informed consent forms for surgery and tissue sampling for scientific investigation. Endometrial tissue specimens were collected from 49 patients who underwent surgery between 2002 and 2005 at The Second Department of Gynecology, Medical University of Lublin, Poland. Forty patients underwent surgery due to EC (cases) and 9 patients due to uterine leiomyomas (controls).

### Macroarray analysis

RNA was isolated according to TRI reagent^®^ protocol (Sigma-Aldrich, USA) and then frozen at −80°C for further preparation. The quality of RNA samples was assessed by gel electrophoresis, and quantitative analysis was performed using a GeneQuant spectrophotometer (Pharmacia, Sweden). Absorption was measured at a 260-nm wavelength, and 1 OD was equivalent to 40 μg of RNA. Reverse transcription of RNA and cDNA labeling were performed with the BD RiboQuant™ RPA system (BD Bioscences-Pharmingen, San Jose, CA, USA) and CDS Primer Mix (BD Biosciences, Clontech, Palo Alto, CA, USA). PCR reaction was then performed using the BD Atlas™ NucleoSpin^®^ extraction kit. In order to perform gene expression profiling, Atlas Human Cancer 1.2 array membranes were used (cDNA Expression Array PT3547-3E; BD Biosciences, Clontech; cat. no. 7851-1). These macroarray membranes contain 1,176 hybridization points for various genes, of which 31 are specific for ECM proteins. Hybridization reactions were performed according to the kit manual, and then membranes were transferred to the exposure cassette BioMax TranScreen HE with Kodak BioMax MS film (Kodak, USA). Exposure times varied and depended on the isotopic activity of the cDNA (between 4 and 7 days). Film development was performed with Kodak liquid developers (Kodak). Results were digitally read with AtlasImage™ software (BD Biosciences, Clontech).

### Statistical analysis

Statistical analyses, including Shapiro-Wilk, Kolmogorov-Smirnov and Lilliefors, Student’s t-test, Chi-squared and Mann-Whitney U tests, and Kruskal-Wallis rank test, were performed with STATISTICA StatSoft v8.0. Numerical measures were summarized as median values and quartiles. Statistical significance was set at α=0.05.

## Results

### Clinicopathological features

The demographic patient data is presented in Table I. We found that patients with stage III ECs were statistically significantly younger compared to those with stage IC and II Ecs (p<0.02 and p<0.03, respectively). We also observed that patients with normal endometrium had their last menstruation at a younger age compared to those with ECs in stages IB and IC (p<0.03 and p<0.04, respectively). A statistically significant difference was also found when comparing the mean age of patients with normal endometrium to that of patients with ECs. This difference was likely due to the younger age of patients who underwent surgery for uterine leiomyomas.

### Macroarray study

**Figure 1:**
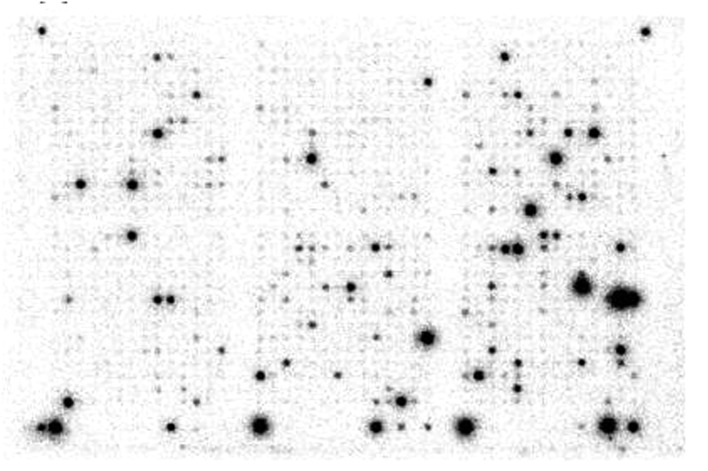
Example of a macroarray membrane after isotopic labeling. Black dots represent expression of the specific genes investigated.

The level of ECM protein gene expression in normal endometrial tissue was compared to expression of these genes in EC specimens. Results of the gene expression in ECs are presented in Table II. In general, statistically significant differences were found in regards to the expression of aggrecan, collagen type VIII chain α1, collagen type XI chain α2, vitronectin, nidogen and tenascin R gene expression stratified by clinical stage according to the International Federation of Gynecology and Obstetrics (FIGO) classification. In all cases analyzed, gene overexpression was noted in advanced stage ECs compared to early stage tumors. Example of a macroarray membrane after isotopic labeling is shown in Fig. 1.

## Discussion

A variety of genetic alterations, in particular those affecting proteins bound to the cell membrane and responsible for cell adhesion and signaling transduction, are responsible for human carcinogenesis. More accurate tools enabling deeper insight into the molecular mechanisms play a crucial role in predicting cancer development and progression. It is of utmost importance to investigate the interactions between different genes, not only changes in expression of individual ones. The ideal tools for gene expression profiling appear to be cDNA macroarrays or microarrays. Along with proper statistical analysis of acquired data it will became the ‘golden standard’.

### Aggrecan

Aggrecan is a major structural component of cartilage, and consists of a protein core of ~220 kDa, with covalently attached glycosaminoglycan side chains, responsible for its unique biochemical properties, integrity and functionality (27,28). Aggrecan is a member of the proteoglycans and influences the adhesive and mitotic activity of cancer cells. Eshchenko *et al* (29) found that expression of another proteoglycan, syndecan, was significantly higher in breast cancer cells compared to that in normal tissues, whereas aggrecan was expressed at the same level in both types of tissues. On the other hand, Japanese researchers (30) reported that mRNA alternative splicing is associated with the malignant transformation of chondrocytes. In the present study, a statistically significant increase in expression was found in cancer cells in stage III according to the FIGO classification. Notably, the expression level was not found to be altered by the histological type of EC (p=0.43). In addition, aggrecan was expressed only in G2 and G3 uterine carcinomas. These results suggest that aggrecan may be an important marker of clinical cancer progression. In order to confirm this result, investigation using more samples should be performed, and the association between aggrecan protein levels and patient survival should also be evaluated.

### Vitronectin

Integrins are heterodimeric glycoproteins that have been found to undergo dynamic temporal and spatial changes in distribution in the endometrium during the menstrual cycle in women. They participate in a wide range of physiological processes, including embryogenesis, wound healing, the immune response and the behavior of malignant cells (31). Moreover, the expression of vitronectin (α_v_β_3_) in melanoma cells has been associated with increased invasiveness (32). Vitronectin is one of the integrins strictly linked to cycle-dependent changes of endometrial cells. Highest expression was found in the luteal phase whether it was not found after menopause or in cancer tissues (33,34). Vitronectin expression was also discovered in patients suffering from endometriosis and infertility. Lessey *et al* (35) investigated 241 endometrium specimens of patients affected by endometriosis between day 19 and 21 of the menstrual cycle and found that lack of β_3_ expression, a subunit of vitronectin, was responsible for the progression of endometriosis (p=0.02). Our results of integrin α_v_β_3_ gene expression, which was observed only in stage III EC cells, suggest that vitronectin plays a role in myoinvasion and increases the ability of cancer cells to metastasize.

### Tenascin

Tenascins are a family of large multimeric ECM proteins present in the connective tissue of vertebrates. To date, four tenascins termed tenascin C, R, X and W have been identified in humans. Contrary to many other ECM proteins, tenascins promote only weak cell adhesion and do not activate cell spreading. They have been classified as anti-adhesive, adhesion-modulating, or even repellent ECM proteins (36). These proteins are also used in bioengineering due to their ability to influence the cell shape (20). Tenascin C (*myotendinous antigen*, *cytotactin*) is synthesized in the central nervous system as well as in peripheral nerves. The most significant expression has been observed during embryogenesis, organo-genesis and during regeneration of mature tissues. Tenascin C is mostly responsible for blocking the adhesion of various cells to fibronectin fibers, which makes cell migration possible in embryogenesis or partially injured nerve regeneration and axon development (36–38).

Tenascin R (*restrictin*, *janusin*) is only synthesized in the central nervous system (39). The biological functions of this protein include the development of new nerve connections and neurite migration in ECM (40,41). We found that the tenascin R gene was expressed only in stage III human uterine carcinomas, which may be an important sign for acquiring an invasive cancer phenotype.

### Nidogen

Nidogen, a glycoprotein, is a binding molecule that links together BM components and is responsible for establishing proper connections within this subtile structure. Nidogen is a single polypeptide chain, folded into two N-terminal globular domains, a C-terminal globular domain and connecting rod-like segments and makes up 3% of extracellular masses (42). There are two separate nidogen particles, encoded by two different genes [nidogen 1 (NID1) and nidogen 2 (NID2)] with molecules of a different size (18,43). Nidogen 2 is more common and it is found mostly in vessels. Both types are responsible for the proper functioning of lungs and blood vessels (44). Additional functions assigned to nidogen include increased cell adhesion ability, neutrophil chemotaxis regulation and necrosis; it also influences neoangiogenesis and trophoblastic development (18,45). To date, altered expression of nidogen has only been observed in the kidney and pancreatic cancer cells (46). According to the functions of this protein and our data, it may be concluded that due to the finding of expression of this gene only in stage III EC cells it may be responsible for increased neoangiogenesis; in addition, it may play a role in defending against infiltration of cancer cells into the BM. Probably, those two processes are synergistic and consist of simultaneous neoangiogenesis in cancer tissues and the necrosis of invaded healthy ECM cells.

### Collagens

The collagen family of structural proteins make up 25% of connective tissue components. There are 25 different types of collagens in the human body responsible for stabilizing the BM and ECM. They play a pivotal role in proper functioning and spatial organization of organs, tissues and blood vessels (15).

Type VIII collagen is produced by intraepithelial cells, keratinocytes and fatty cells. Increased concentrations of this collagen were found in migrating smooth muscle cells and intraepithelial vessel cells. It is hypothesized that it increases the production of metalloproteinases (MMPs), which increase cell migration ability (47,48). Collagen type VIII mRNA overexpression was observed in the region surrounding new blood vessel formation (49).

Type XI collagen is a fibrillar protein forming the scaffold for skin, bones, tendons and ligaments (15). In 2008, Halsted *et al* (50) published the results of an immunohistochemical study of 72 breast cancer samples and healthy tissues from the same patients. They found decreased expression of α chain collagen XI in cancer tissues when comparing to the expression in healthy tissues (p<0.01) and in metastasis compared to primary tumors (p=0.01). Based on these observations, the authors concluded that decreased expression of collagen may be helpful in identifying patients presenting with metastases.

Presently, we found increased expression of α types VIII and XI collagens in EC tissues compared to levels in normal tissues, yet statistical significance was found only between normal and stage III ECs.

Finally, the results of our macroarray study of ECM gene expression profiles may shed some light on the investigational methods for cancer prognosis. Gene expression levels of the above-mentioned proteins are likely to be important markers of clinical cancer progression, metastasis formation and neoangiogenesis. These functions are responsible for cancer progression and, if investigated further, may be additional diagnostic and prognostic tools for women suspected of or suffering from ECs.

## Figures and Tables

**Table I:** Patient data stratified by the FIGO staging system.

FIGO stage	Patients, n (%)	Age ± SD (years)	Parity ± SD (n)	FM ± SD	LMP ± SD
IB	16 (32.6)	61.6±10.24	2.1±1.09	13.9±1.84	51.2±3.36
IC	14 (28.6)	69.1±8.99	2.9±2.27	14.8±1.75	51.3±3.24
II	5 (10.2)	67.7±4.99	2.0±0.82	14.5±1.00	51.5±2.38
III	5 (10.2)	52.2±3.77^a^	1.2±1.26	14.0±0.82	50.7±2.22
Normal endometrium	9 (18.4)	50.1±7.56^b^	2.0±1.12	14.3±1.66	47.6±3.03^c^

FM, first menstruation; LMP, last menstrual period.

a Statistically significant younger patients with EC in stage III compared to patients at stage IC (p<0.01) and stage II (p<0.01), respectively.

b Statistically significant younger patients with normal endometrium compared to patients with EC at stage IB (p<0.01), stage IC (p<0.01) and stage II (p<0.01), respectively.

c Statistically significant lower age of LMP in the group with normal endometrium compared to women with EC at stage IB (p<0.04) and stage IC (p<0.04), respectively.

**Table II:** Statistical descriptive analysis of the investigated samples according to FIGO classification.

Gene	FIGO stage	Median	Lower quartile	Upper quartile	Range		Analysis	
	
					Min	Max	H	P-value
Aggrecan	I	0.00	0.00	0.00	0.00	0.22	6.60	0.04^a^
	II	0.00	0.00	0.00	0.00	0.18		
	III	0.07	0.00	2.24	0.00	2.89		
Collagen type VIII α1	I	0.00	0.00	0.00	0.00	0.16	9.06	<0.01^a^
	II	0.00	0.00	0.00	0.00	0.00		
	III	0.00	0.00	0.39	0.00	0.46		
Collagen type XI α2	I	0.00	0.00	0.00	0.00	0.00	14.36	<0.01^a^
	II	0.00	0.00	0.00	0.00	0.00		
	III	0.00	0.00	0.37	0.00	1.11		
Vitronectin	I	0.00	0.00	0.00	0.00	0.00	14.36	<0.01^a^
	II	0.00	0.00	0.00	0.00	0.00		
	III	0.00	0.00	0.34	0.00	0.53		
Nidogen	I	0.00	0.00	0.00	0.00	0.00	7.00	0.03^a^
	II	0.00	0.00	0.00	0.00	0.00		
	III	0.00	0.00	0.00	0.00	1.11		
Tenascin R	I	0.00	0.00	0.00	0.00	0.00	7.00	0.03^a^
	II	0.00	0.00	0.00	0.00	0.00		
	III	0.00	0.00	0.00	0.00	1.01		

a Statistically significant gene expression difference in endometrial cancer tissues compared to normal endometrium (Kruskal-Wallis rank test).
